# Fate After the Mustard Procedure for d-Transposition of the Great Arteries: Impact of Age, Complexity, and Atrial Tachyarrhythmias: A Single Center Experience

**DOI:** 10.1007/s00246-023-03241-7

**Published:** 2023-07-28

**Authors:** Ulrich Krause, Sophie Theres Teubener, Matthias J. Müller, Heike E. Schneider, Thomas Paul

**Affiliations:** Department of Pediatric Cardiology, Intensive Care Medicine and Neonatology, University Medical Center, Georg-August-University Göttingen, Robert-Koch-Str. 40, 37099 Göttingen, Germany

**Keywords:** Transposition of the great arteries, Mustard, Heart failure, Sudden cardiac death, ICD

## Abstract

Patients with dextro transposition of the great arteries (d-TGA) after atrial switch procedure are at risk to develop heart failure and arrhythmias during long-term follow-up. The present study aims to add knowledge on the fate of subjects after Mustard procedure during long-term follow-up into adulthood. A single center, retrospective chart review analysis was conducted. All subjects who had Mustard-type atrial switch procedure between 1969 and 1994 at our institution were included. A total of 92 subjects were included. Early postoperative death was reported in 2 subjects. Long-term follow-up was available in 49 survivors. Of those, 6 individuals died during further follow-up. Sudden cardiac death was the most prevalent cause for fatal outcome. Mortality during long-term follow-up was associated with the presence of additional cardiovascular malformations (complex d-TGA). Sinus node dysfunction was observed in 65% of the patients and atrial tachyarrhythmias were common in adult survivors (63%). Implantation of a pacemaker or a cardioverter defibrillator was required in 31% and 45% of those surviving into adulthood. Complications were frequently observed during follow-up after either pacemaker or cardioverter defibrillator implantation (43%) with lead failure being the most frequent complication. The aging population of patients after Mustard procedure is facing challenging problems mainly resulting from a failing systemic right ventricle, presence of associated cardiac malformations and the presence of atrial baffles associated with relevant atrial scars. Age, associated cardiac malformations, and atrial tachyarrhythmias seem to play a major role in determining the fate of patients with d-TGA after atrial switch procedures.

## Introduction

Dextro-transposition of the great arteries (d-TGA) is the second most common cyanotic congenital heart disease, comprising 2–3% of all congenital heart defects and leading to death in almost all babies affected within the first year of life in the absence of additional cardiac malformations. Introduction of the atrial switch procedure by Senning in 1959 [1] and Mustard in 1964 [2] allowed long-term survival of individuals with d-TGA into adulthood. Though the atrial switch procedure dramatically improved survival of patients with d-TGA, several significant sequelae became apparent with increasing age. During mid-term follow-up a significant portion developed sinus node dysfunction and atrial tachyarrhythmias. In patients > 30 years of age systemic right ventricular dysfunction and tricuspid valve regurgitation became apparent leading to heart failure. Systemic right ventricular dysfunction, tricuspid regurgitation, and atrial tachyarrhythmias were identified as risk factors for ventricular tachyarrhythmias and ultimately sudden cardiac death (SCD) decades after the atrial switch procedure [3–5].

Though the arterial switch operation (ASO) replaced the atrial switch operation since the 1980s patients after atrial switch procedure required even increased attention for heart rhythm and heart failure management as they became continuously older. Several reports on specific problems like SCD or atrial arrhythmias in this unique population were published in the past [3, 6, 7], however, data on the overall course of individuals after atrial switch procedure is still sparse [5, 8, 9].

The aim of this study was to analyze the long-term outcome of patients with d-TGA after Mustard-type atrial switch procedure from a large tertiary adult congenital heart disease (ACHD) center covering follow-up data from more than half a century.

## Patients and Methods

A longitudinal, retrospective single center analysis of all patients with d-TGA who had had Mustard procedure at our institution between 1969 and 1994 was performed. As the Mustard technique was used in all subjects for atrial switch at our institution, patients after Senning-type atrial switch procedures are not covered in the present study. Data were acquired from the patients` hospital records and follow-up was continued until February 2022, covering up to 53 years of individual follow-up.

This study was approved by the institutional review board and all subjects provided written consent for scientific use of their data.

Primary endpoints were all cause mortality and heart transplantation, secondary endpoints were severity of heart failure, brady- and tachyarrhythmias and atrial baffle obstruction and/or leakage. Demographic data at the time of atrial switch procedure and procedural data were recorded from the patients´ hospital records.

‘Complex TGA’ was defined as d-TGA with additional relevant cardiac malformations other than atrial septal defect (ASD) or patent ductus arteriosus (PDA). All other patients were referred as ´simple TGA´. Sinus node dysfunction was defined as sinus bradycardia (or any other slow escape rhythm in subjects with complete loss of sinus node function) and a mean heart of < 50 beats per minute on 24 h Holter monitoring. Additionally, subjects with sinus pauses of > 3 s were diagnosed to have sinus node dysfunction, irrespective of mean heart rate. Atrial arrhythmias (either reentrant or focal) were defined as any tachyarrhythmia originating from the systemic venous (“right”) or pulmonary venous (“left”) atrium and not from the sinus node. Intraatrial reentrant tachycardia was diagnosed as an intraatrial circus movement tachycardia inducible by programmed atrial stimulation or burst atrial stimulation and focal atrial tachycardia was diagnosed as atrial tachycardia originating from a single site other than the sinus node. Demographic data as well as detailed information about the surgical procedure, arrhythmias, and follow-up were obtained. Early postoperative death was defined as death for any reason within 3 months after the Mustard operation. Symptoms according to the NYHA classification reported during the last follow-up visit were used for analysis.

Patients surviving into adulthood were regularly followed on an annual basis at our ACHD outpatient clinic.

### Statistics

Statistical analysis was performed using SPSS® 28.0 software (IBM, Armonk, USA). Numerical data are presented as median and interquartile range (IQR) unless otherwise indicated. Differences in categorical variables were calculated by Chi-square or Fischer´s exact test where appropriate. Differences in parametric data were calculated by Mann–Whitney-*U*-test and Kruskal–Wallis-test where appropriate. Pearson coefficient was used for bivariate analysis for correlation. Analysis of association of nominal parameters was performed using Phi and Cramer-*V* test. Kaplan–Meier analysis was performed for analysis of event-free survival. Statistical significance was set at *p* < 0.05.

## Results

### Patient Characteristics

**Fig. 1:**
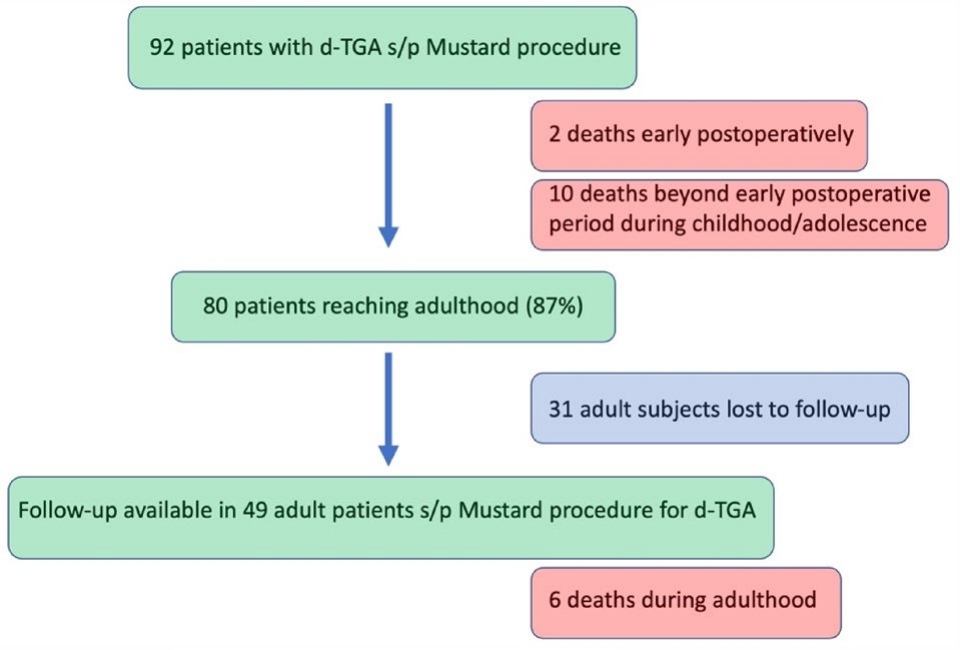
Fate of 92 subjects with d-TGA undergoing the Mustard procedure (*d-TGA* = dextro-transposition of the great arteries)

A total of 92 consecutive subjects (26% female) with dextro-transposition of the great arteries (d-TGA) after Mustard procedure were enrolled into the study. At the time of Mustard procedure, median patient age was 10 (IQR 7–21) months and median body weight was 7.3 (IQR 6.2–9) kg. Complex d-TGA had been present in 23/92 (25%) individuals. Surgical systemic-to-pulmonary shunt palliation prior to Mustard procedure had been performed in 8/92 (9%) patients. Early postoperative death was reported in 2/92 individuals (2%). Figure 1 gives an overview on the fate of all subjects initially enrolled.

### Baffle Obstruction and Baffle Leakage

From the cohort of 90 subjects initially surviving beyond the early postoperative period, a total of 34 patients (38%) developed either isolated baffle obstruction, isolated baffle leakage or combined baffle stenosis and leakage. Isolated baffle obstruction was found in 19/90 (20%) subjects while isolated leakage was reported in 5/90 (6%) individuals. Combined baffle obstruction and baffle leakage was reported in 10/90 (11%) patients. Baffle obstruction and/or baffle leakage were not associated with the time of the Mustard procedure as surrogate parameter for surgeons´s experience/learning curve (*p* = 0.5).

Surgical baffle revision during childhood was performed in 9/34 (26%) patients. Catheter-based treatment with implantation of one or more stents for either baffle obstruction or combined obstruction/leakage was performed in 16/34 (47%) subjects while balloon angioplasty of isolated baffle obstruction was performed in 2/34 (6%) individuals while catheter-based device closure of isolated baffle leakage was required in another patient (1/34; 3%). Baffle problems requiring surgery were identified within the first year after Mustard procedure (median time to diagnosis 5.3 months following the Mustard procedure). In contrast, baffle stenosis and/or leakage requiring catheter-based treatment was diagnosed exclusively in adult patients (median time to diagnosis 31.4 years following the Mustard procedure). In the remaining 6 patients (18%) no interventional or surgical intervention was deemed necessary.

Presence of baffle obstruction or baffle leakage was not associated with heart failure or mortality during long-term follow-up (*p* = 0.9).

### Survival and Follow-Up

From the initial cohort of 92 individuals two subjects with complex d-TGA died early postoperatively (2%). Beyond the early postoperative period, 10 subjects died for various reasons before reaching adulthood (sudden cardiac death *n* = 4; heart failure *n* = 1; myocarditis *n* = 1; intracranial hemorrhage *n* = 1; pulmonary thromboembolism *n* = 1; unknown *n* = 2, Table 1). The 4 subjects who died suddenly before reaching adulthood were not systematically evaluated for their individual risk for sudden cardiac death. The exact cause of death had not been evaluated in those patients, however, sudden cardiac death was diagnosed, as no other conditions possibly resulting in sudden death (like epilepsy) were known in those patients. No postmortem studies were available as in those 4 patients natural death was declared. However, in those 4 subjects, univariate risk assessment showed association of sudden cardiac death with the presence of atrial tachyarrhythmias (*p* = 0.001) and the presence of a pacemaker system (*p* = 0.001, Table 3). Of the remaining 80 patients, follow-up data was available from 49 subjects, whereas 31 individuals were lost to follow-up (Fig. 1). Those 49 patients (22% female) formed the cohort of adult patients for further analysis with a median follow-up of 34 (IQR 27–39) years (Table 1). Three of those individuals were followed ≥ 50 years with a maximum follow-up of 53 years. Median age at last follow-up was 39 (IQR 36–50, *n* = 49) years and median body mass index (BMI) was 24.7 kg/m^2^ (IQR 22.8–27.9 kg/m^2^, *n* = 49).

**Table 1:** Main characteristics of patients surviving into adulthood (*d-TGA* = dextro transposition of the great arteries; FU = follow up; *ICD* = implantable cardioverter defibrillator)

Patients, *n*	49
Complex d-TGA, *n* (%)	11 (22.4)
Median age @ last FU (years)	39
Median BMI @ last FU (kg/m2)	27.4
Sinus node dysfunction, *n* (%)	32 (65)
Atrial tachycardias, *n* (%)	31 (63)
Pacemaker, *n* (%)	15 (31)
ICD (total), *n* (%)	22 (45)
ICD (primary prevention), *n*	20
ICD (secondary prevention), *n*	2
Heart transplantation, *n*	1
Death, *n* (%)	6 (12)

### Late Mortality

**Fig. 2:**
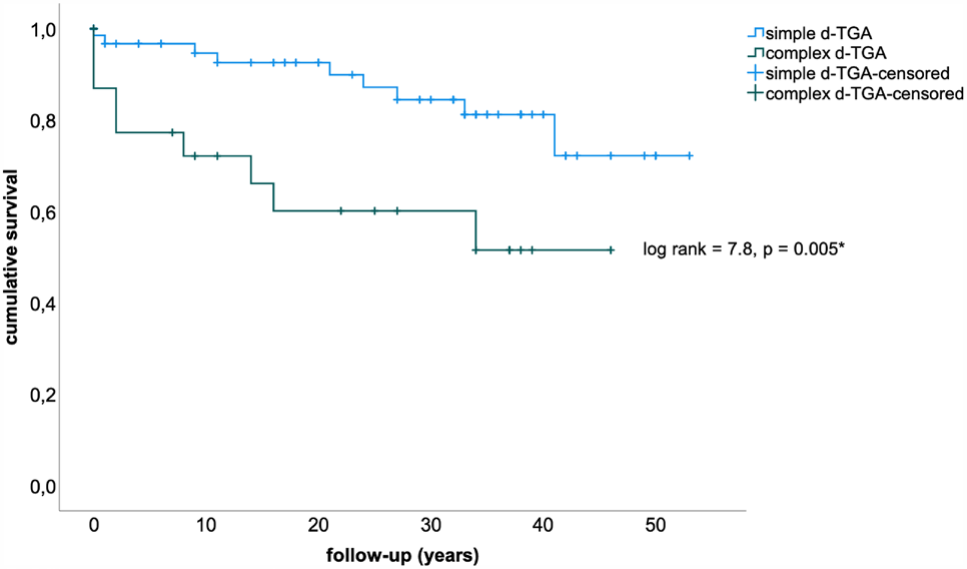
Kaplan–Meier curve showing significantly shorter survival of patients with complex d-TGA (dark green line) compared to those with simple d-TGA (blue line; *d-TGA* = dextro-transposition of the great arteries)

During long-term follow-up 6/49 (12%) survivors of the Mustard procedure died due to sudden cardiac death (*n* = 4), heart failure (*n* = 1), and early after combined heart–lung transplantation (*n* = 1). Table 2 summarizes causes of death in patients after the Mustard procedure for d-TGA. Survival was significantly impaired for patients with complex d-TGA compared to simple d-TGA (29 vs. 45.4 years, log Rank 7.8, *p* = 0.005, Fig. 2).

**Table 2:** Causes of death in 18/92 (20%) patients (*HLTX* = combined heart and lung transplantation; *SCD* = sudden cardiac death)

Cause of death	during childhood/adolescence (< 18 years, *n* = 12)	during adulthood (≥ 18 years, *n* = 6)
SCD	4	4
End stage heart failure	1	1
Myocarditis	1	
Pulmonary thromboembolism	1	
Post HLTX		1
Intracranial hemorrhage	1	
Early postoperatively	2	
Unknown	2

**Fig. 3:**
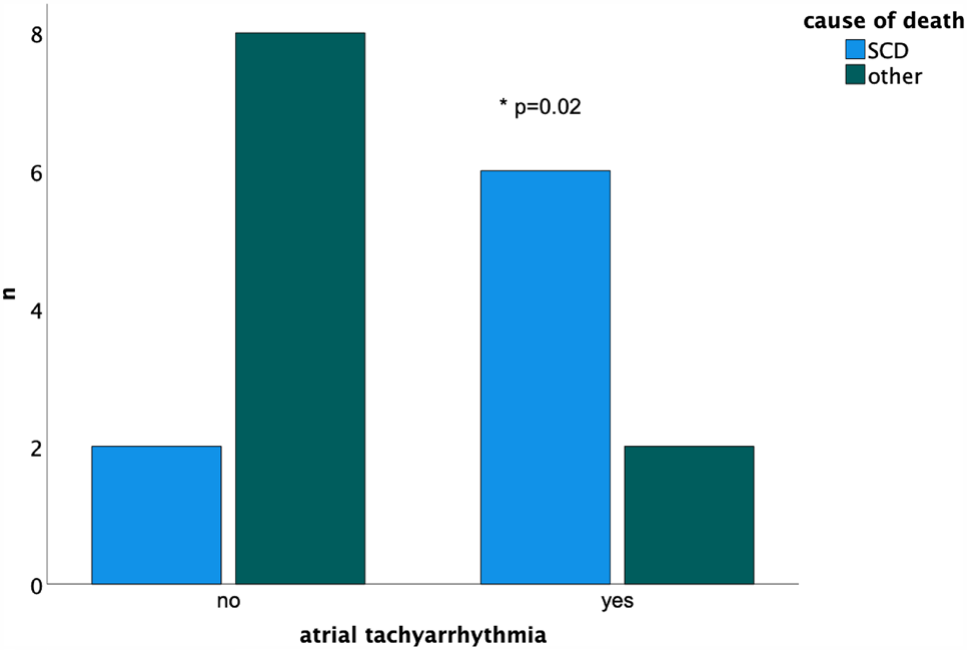
History of atrial tachyarrhythmia was significantly associated with sudden cardiac death (*SCD*) during follow-up after Mustard procedure

Long-term survivors after the Mustard procedure had significantly shorter QRS complex duration (110 ms, IQR 100–140 ms, *n* = 43 vs. 140 ms, IQR 119–183 ms, *n* = 6; *p* = 0.04). SCD was associated with a history of atrial tachycardias in survivors after the Mustard procedure (*p* = 0.02, Fig. 3, Table 3), whereas age was not associated with total mortality and SCD (*p* = 0.09).

**Table 3:** Univariate risk assessment for factors associated with sudden cardiac death in subjects after Mustard procedure during childhood and adolescence (“early era”) and in those surviving into adulthood (“late era”)

	early era (*n*)	p	late era (*n*)	p
AT	6	***0.001***	31	***0.02***
ns VT	–	–	16	0.74
PM	7	***0.001***	12	0.98
ICD	–	–	22	0.41
RV EF < 35%	–	–	1	0.77

Significant p-values are given in bold italics

The presence of atrial tacharrhythmias and pacemakers was associated with an increased risk for sudden cardiac death. (AT = atrial tachyarrhythmia; ICD = implantable cardioverter defibrillator; ns VT = non-sustained ventricular tachycardia; PM = pacemaker; RV EF = right ventricular ejection fraction)

### Arrhythmias

Heart rhythm status was monitored by annual 24 h Holter monitoring and interrogation of pacemakers or implantable cardioverter defibrillators every 6 months, if appropriate. At last follow-up visit sinus rhythm was preserved in 17/49 (35%) adult patients. The remaining 32 subjects had sinus node dysfunction. Thereof, 28/49 (57%) subjects had indication for permanent pacing due to sinus node dysfunction (see below). Complete atrioventricular (AV) block requiring permanent ventricular pacing was reported in 4/49 (8%) subjects including postoperative AV block after closure of ventricular septal defect in 3 patients and after radiofrequency catheter ablation of AV nodal reentrant tachycardia in 1 individual.

Atrial tachyarrhythmias were observed in 31/49 patients during follow-up into adulthood (63%). Sinus node dysfunction was not linked to occurrence atrial tachyarrhythmias (*p* = 0.34), while baffle obstruction and/or baffle leakage were associated with atrial tachyarrhythmias (*p* = 0.01). Atrial tachyarrhythmias were strongly associated with length of postoperative follow-up after Mustard procedure (eta = 0.83). Catheter ablation of atrial tachyarrhythmias was performed in 22/31 (71%) individuals. Catheter ablation of atrial tachyarrhythmias was not associated with favorable outcome with respect to mortality (*p* = 0.4). In those 2 subjects experiencing inadequate ICD discharge due to rapidly conducted intraatrial reentrant tachycardia (see below), rapidly conducted intraatrial reentrant tachycardia in fact was indication for catheter ablation. No inadequate ICD discharges were observed after catheter ablation.

Non-sustained ventricular tachycardia (VT) was documented during Holter monitoring or on pacemaker (PM)/implantable cardioverter defibrillator (ICD) telemetry in 16/49 subjects (33%). VT was moderately associated with length of postoperative follow-up after Mustard procedure (eta = 0.75). No association between the occurrence of atrial and ventricular tachyarrhythmias during follow-up could be detected.

### Cardiac Implantable Electronic Devices

At last follow-up, 15/49 (31%) patients had a pacemaker system implanted for sinus node dysfunction in 11 and AV block in 4 patients. 22/49 (45%) individuals had had implantation of an ICD. Transvenous dual chamber ICD implantation was performed in 21/22 (95%) subjects. A single individual had a subcutaneous ICD (EMBLEM™ S-ICD, Boston Scientific, Marlborough, USA) implanted due to lead-associated thrombosis of the superior vena cava and the superior Mustard baffle.

Indication for ICD implantation was primary prevention of SCD in 20/22 (91%) patients, whereas secondary prevention was given in 2/22 (9%) subjects. Table 4 provides detailed information on primary prevention indications. 17/22 (77%) of ICD patients had permanent pacing due to sinus node dysfunction while 1 individual (5%) had permanent pacing for complete AV block. A total of 5 ICD discharges in 5/22 (23%) ICD patients were noted. Of those, 3 (60%) were inappropriate discharges due to rapidly conducted intraatrial reentrant tachycardia (*n* = 2) and lead failure (*n* = 1) while appropriate ICD discharges occurred in 2/22 patients (9%) 27 and 48 years after Mustard procedure, respectively. Both subjects with appropriate ICD therapies were male and had their ICD for primary prevention of SCD. One of those 2 patients had complex d-TGA associated with coarctation of the aorta and ventricular septal defect s/p surgical repair. This particular patient additionally suffered from coronary artery disease and peripheral artery disease. The second patient had simple d-TGA. Both subjects had severely impaired RV function (EF < 35%) and were treated with betablockers and ACE inhibitors at the time of appropriate ICD discharge.

**Table 4:** Primary prevention indications for ICD implantation in 20 subjects (*ns VT* = non-sustained ventricular tachycardia; *inducible VT/VF* = ventricular tachycardia inducible on programmed ventricular stimulation; *RV EF* = right ventricular ejection fraction; *RV ischemia* = right ventricular ischemia)

Indication	*n* (%)
ns VT	8 (40)
inducible VT/VF	5 (25)
RV EF < 35%	6 (30)
RV ischemia	1 (5)

Complications after pacemaker or ICD implantation were frequent as observed in 15/35 (43%) individuals. Isolated lead failure was most common and was observed in 9/15 subjects with accompanying lead infection in 1 patient. Lead-related obstruction/thrombosis of the superior vena cava and the superior Mustard baffle was noted in 4/15 subjects while another 2/15 patients had infection of their endocardial ICD-system. One of those subjects had lead-related infectious endocarditis, the other one had pocket infection 6 months after implantation. In both individuals, the ICD generator and all endocardial leads were extracted and a new endocardial ICD-system was implanted after sufficiently long antibiotic treatment. In both subjects, leads of the new ICD-system were implanted via the contralateral cephalic vein and a new generator pocket was created.

### Heart Failure

At last follow-up visit, median heart failure severity score according to the New York Heart Association (NYHA) classification was 2 (IQR 1–2, *n* = 49). Severity of heart failure was not associated with presence of complex d-TGA vs. simple d-TGA (*p* = 0.54). Age at Mustard procedure was positively correlated with NYHA severity score (*p* = 0.04), i.e., the older patients´ age at the time of Mustard procedure, the more severe heart failure symptoms were present at last follow-up. Data on maximum workload during exercise stress test at last follow-up was available in 42/49 (86%) patients. Median maximum workload was 1.8 W/kg (IQR 1.5–2.1 W/kg). Data on maximum oxygen uptake during stress test was available in 35/49 (71%) patients. Median maximum oxygen uptake was 21.4 ml/kg*min (IQR 19.6–25 ml/kg*min). As with NYHA classification severity score, workload/kg and maximum oxygen uptake did not differ between patients with complex d-TGA and those with simple d-TGA (*p* = 1.0 and *p* = 0.6, respectively). No correlation between age at Mustard procedure and results of exercise stress test could be found.

Right ventricular filling pressure (RVedP) was measured during cardiac catheterization in 41/49 (84%) individuals with a median of 10 (7–12) mmHg. There was no difference of RVedP between patients with complex d-TGA and those with simple d-TGA (*p* = 0.64). Duration of follow-up and patients´ age at last follow-up were not related with RVedP (*p* = 0.28 and *p* = 0.32, respectively).

Magnetic resonance imaging (MRI) data on performance of the systemic right ventricle was available in 12/49 (25%) subjects. Median right ventricular enddiastolic volume index (RVedVi) was 127 (IQR 102–165) ml/m^2^ and median right ventricular ejection fraction (RV EF) was 48 (IQR 38–50)%.

### Heart Failure Therapy

At last follow-up visit, angiotensin-converting enzyme (ACE) inhibitors/angiotensin II receptor type 1 (AT_1_) antagonists were prescribed in 31/49 (63%) patients for treatment of reduced RV ejection fraction/heart failure. Additional heart failure medication included ß-blockers prescribed in 26/49 (53%) patients, aldosterone antagonists in 11/49 (22%) subjects, and diuretics in 12/49 (25%) individuals.

### Heart Transplantation

Heart transplantation for end stage heart failure due to severely impaired right ventricular function was performed in one individual after myocardial infarction at age 47. Combined heart–lung transplantation due to accompanying severe pulmonary artery hypertension was performed in another patient who died early after surgery due to septicemia.

### Cardiovascular Co-Morbidities

Systemic arterial hypertension was diagnosed in 6/49 (12%) individuals and pulmonary artery hypertension was found in 4/49 (8%) subjects. While systemic arterial hypertension was not associated with mortality, pulmonary artery hypertension showed a significant association with fatal outcome (*p* = 0.024).

## Discussion

**Fig. 4:**
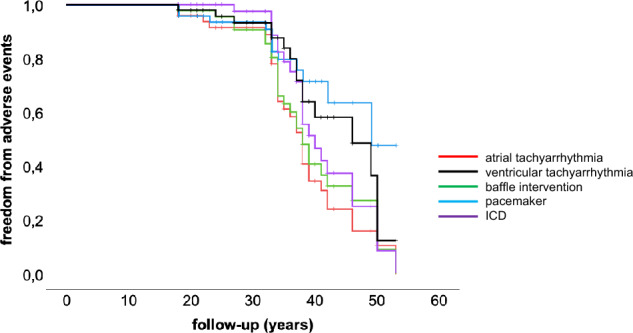
Event-free survival of adult patients (*n* = 49) following the Mustard procedure (*ICD* = implantable cardioverter defibrillator)

The present study analyzes data from a group of patients with d-TGA after atrial switch procedure exclusively applying the Mustard technique from a single tertiary ACHD center. The study confirms the fact, that those individuals and their physicians have to cope with a variety of serious and challenging problems during mid- and long-term follow-up, primarily resulting from the presence of a morphologically right ventricle serving as systemic ventricle and from multiple suture lines within the atrial myocardium serving as substrate for atrial tachyarrhythmias years and even decades after surgery. The main results from the present study are: (1) fatal outcome was associated with the presence of complex d-TGA, atrial tachyarrhythmias and pulmonary artery hypertension; (2) age was a matter of concern, as the prevalence of adverse events including atrial and ventricular tachyarrhythmias, need for either a pacemaker or an ICD, baffle intervention and death increased with age (Figs. 2 and 4); (3) a relevant number of patients required either pacemaker implantation or ICD implantation during long-term follow-up and 4) inappropriate ICD-discharges were a matter of concern in this unique patient population.

### Morbidity and Mortality

Early death within the first month after surgery was rare in our cohort while accounting for up to 20% in reports from other institutions [10]. The reason for this low rate of early postoperative death is not entirely clear. However, our institution was the one with the highest experience of atrial switch procedures applying the Mustard technique in those days and operator´s experience and a highly experienced congenital heart team might have been significantly contributed to this favorable numbers. A significant number of patients was lost to follow up because they were referred from outside the catchment area of our institution for the Mustard procedure, as our institution was one of the few institutions in Germany doing Mustard procedures in those days. After discharge from the hospital postoperatively, further follow up of those patients was performed by pediatric cardiologists and adult congenital cardiologists, commonly referring patients to other institutions. Because of this and because patients changed their primary care physician, it was impossible to retrieve follow-up data on those subjects.

In our cohort morbidity within the first 3 decades following the Mustard procedure was sparse, followed by significant morbidity in the mid-thirties (Fig. 4). Patients with complex congenital heart disease > 30 years of age have been reported to be at a constantly growing risk for fatal outcome [11]. The additional burden of associated malformations in subjects with complex d-TGA probably played a pivotal role determining long-term outcome as this subgroup had higher mortality and shorter survival during follow-up as previously reported [12]. The burden of additional surgery like VSD closure or coarctation repair might well be one reason for worse outcome.

During long-term follow-up, sudden cardiac death was the most common reason for a fatal outcome. Atrial tachyarrhythmias were significantly linked to SCD. Atrial tachycardia with high rate AV conduction leading to ventricular ischemia is the proposed pathophysiology for sudden cardiac death in this unique patient population [13]. Thus, atrial tachyarrhythmias should be rigorously treated by catheter ablation to reduce risk of stroke and heart failure and to minimize risk for sudden cardiac death realizing that repeat catheter ablation due to tachycardia recurrence or new atrial tachycardias is often required in those individuals [14, 15].

### Heart Failure

It is of note, that despite impaired systemic RV function, heart failure medication was prescribed to only roughly half of our patients. It should be emphasized that a considerable number of ACHD patients have decreased aerobic capacity without any or only neglectable symptoms [16]. Quantifying systolic RV function by echocardiography is unreliable while quantifying RV ejection fraction by MRI is the gold standard. However, in our cohort, presence of either pacemaker or ICD systems in a relevant number of adult patients after the Mustard procedure precluded more patients from having MRI scans as reported before [5, 8]. Therefore, assessment of heart failure in a considerable part of the young ACHD population, including those with d-TGA after atrial switch repair, is challenging. Paucity of overt heart failure symptoms might be the primary reason for infrequent use of heart failure medication in those patients.

As myocardial ischemia aggravated either by sinustachycardia or by rapidly conducted AT has been hypothesized as major risk factor for life threatening ventricular tachyarrhythmias in subjects with d-TGA after atrial switch procedure [13], therapy with betablockers in order to reduce maximum heart rate and myocardial oxygen consumption is highly recommended. In addition afterload-reducing agents like ACE inhibitors to reduce right ventricular filling pressure and to increase cardiac output in patients with significant tricuspid regurgitation should become standard after atrial switch procedure, irrespective of heart failure symptoms [17].

### Atrial and Ventricular Tachyarrhythmias, ICD Therapy

Intraatrial reentrant tachycardias are of major concern in the adult d-TGA population after atrial switch procedures, as those individuals commonly exhibit unimpaired atrioventricular conduction. Therefore, sustained atrial tachycardias increase the risk for cerebral thromboembolism and progression of congestive heart failure. Rapid atrioventricular conduction of atrial tachyarrhythmias may result in profound myocardial ischemia despite normal coronary arteries in these patients with impaired function of the hypertrophied systemic right ventricle [13, 18]. Due to rapid AV conduction of atrial tachycardia patients with an ICD are at increased risk of inappropriate ICD therapies [19–21]. In our cohort, inappropriate ICS shocks were delivered due to rapidly conducted AT and lead fracture. Therefore, rigorous treatment of AT, decent ICD programming, and close remote monitoring of lead performance seems to be mandatory. A recently published study did not report a benefit from remote device monitoring with respect to number of inappropriate ICD discharges in an unselected patient population with structurally normal hearts [22]. There is, however, no data on remote device monitoring in ACHD patients. Therefore, it remains debatable, whether results from a population without congenital heart disease can be transferred to the ACHD population.

## Conclusion

The aging population of patients after Mustard procedure is increasingly facing challenging problems mainly resulting from a failing systemic right ventricle, presence of associated cardiac malformations, and redirection of systemic venous as well as pulmonary venous blood through atrial baffles associated with relevant atrial scars. Age, associated cardiac malformations, and atrial tachyarrhythmias seem to play a major role in determining the fate of patients with d-TGA after atrial switch procedures. Though previous studies suggested models for risk stratification in subjects with a systemic RV and especially in patients with d-TGA after atrial switch procedure, a validated algorithm for risk estimation in those patients is still missing. However, age, presence of associated cardiac malformations, and a history of atrial tachyarrhythmias seem to be of pivotal significance for the fate of subjects after atrial switch procedure.

### Limitations

The present study is limited by its single center character and the number of patients lost to follow-up. Analyzing exclusively patients after Mustard-type atrial switch repair may limit transfer of results and conclusions to patients after a Senning-type operation.
